# Enthalpies of formation of Cd–Pr intermetallic compounds and thermodynamic assessment of the Cd–Pr system

**DOI:** 10.1016/j.calphad.2014.06.005

**Published:** 2014-12

**Authors:** Thomas L. Reichmann, Klaus W. Richter, Simona Delsante, Gabriella Borzone, Herbert Ipser

**Affiliations:** a Department of Inorganic Chemistry (Materials Chemistry), University of Vienna, 1090 Wien, Austria; b Dipartimento di Chimica e Chimica Industriale, Università di Genova, 16146 Genova, Italy

**Keywords:** CALPHAD, Phase diagram Cd–Pr, Enthalpy of formation, Alloys

## Abstract

In the present study standard enthalpies of formation were measured by reaction and solution calorimetry at stoichiometric compositions of Cd_2_Pr, Cd_3_Pr, Cd_58_Pr_13_ and Cd_6_Pr. The corresponding values were determined to be −46.0, −38.8, −35.2 and −24.7 kJ/mol(at), respectively. These data together with thermodynamic data and phase diagram information from literature served as input data for a CALPHAD-type optimization of the Cd–Pr phase diagram. The complete composition range could be described precisely with the present models, both with respect to phase equilibria as well as to thermodynamic input data. The thermodynamic parameters of all intermetallic compounds were modelled following Neumann–Kopp rule. Temperature dependent contributions to the individual Gibbs energies were used for all compounds. Extended solid solubilities are well described for the low- and high-temperature modifications of Pr and also for the intermetallic compound CdPr. A quite good agreement with all viable data available from literature was found and is presented.

## Highlights

• Δ*fH*° was determined from Cd_2_Pr, Cd_3_Pr, Cd_58_Pr_13_ and Cd_6_Pr.
• Thermodynamic assessment of the Cd–Pr phase diagram.
• CALPHAD-type optimization of the complete Cd–Pr system is presented.
• Temperature dependent contributions to the individual Gibbs energies were used.

## 1 Introduction

In the last decades, the question of how to satisfy the ever increasing demand of energy has become most pressing for countries with high economic growth. The utilization of nuclear energy is sometimes inevitable for nations belonging to the group of developing and emerging countries. For an efficient use of nuclear energy, these countries have to establish strategies comprising an optimized reprocessing routine of spent nuclear fuels as well as an adequate waste management on the back-end of their nuclear fuel cycle. Indeed, low-level and intermediate-level radioactive waste is currently stored in interim storage facilities or deposited in geological repositories. Solutions for high-level waste are currently still in a planning stage and thus this type of waste is actually stored on-site.

Focusing onto reprocessing of nuclear waste, this is currently practiced by means of solvent extraction of actinides using tributyl phosphate (TBP), known as hydro-metallurgical technique or aqueous reprocessing (e.g., PUREX), respectively. Unfortunately, this technique deals with several problems like radiation and temperature instability of the various solvents used in the process. In addition, a huge amount of liquid waste is produced when applying PUREX or related processes. It is therefore reasonable to investigate an alternative type of reprocessing, called the pyro-metallurgical technique. The latter technique is not dealing with the major disadvantages of the aqueous methods, as outlined by Olander [1], and it is described repeatedly in literature, see e.g., Refs. [2], [3], [4], [5].

In particular, electro-transport and reductive extraction is applied to separate actinides and lanthanides from high-level radioactive waste inside an “electro-transporter” cell. The respective electrochemical vessel contains a liquid metal pool at the bottom, covered by a molten salt solution serving as electrolyte. One basket anode, containing chopped nuclear fuels, and at least two cathodes are inserted into the liquid salt. During the process especially uranium, plutonium and minor actinides are transported whereas rare earth elements, alkaline and alkaline earth elements remain in the liquid salt. Additional reductive agents like Li are added to the salt which promotes the extractability of rare earth (RE) elements into the liquid metal pool at the bottom by forming intermetallic compounds. Moriyama et al. [6], [7] determined that the separation factors, which are an indicator for extractability, are quite different between actinides and lanthanides. In principle, actinides have the higher affinity for extraction into a metal phase, a fact that is desirable, considering the chemical similarity to lanthanides. The extraction behaviour of different elements between a molten chloride salt phase and a liquid metal strongly depends on the standard free energy of formation of the corresponding chlorides as well as on the activity coefficients of the extracted elements in the respective intermetallic compounds. Thus the separation factors are strongly influenced by the employed liquid metal which is preferentially Cd [8]. Therefore, a detailed knowledge of the respective Cd–RE phase diagrams as well as of the thermodynamic stabilities of the corresponding intermetallic compounds is of great importance. This was the reason for initiating a series of thermodynamic and phase diagram studies of different Cd–RE systems (cf. Refs. [9], [10], [11]).

It was the aim of the present study to perform a CALPHAD-type optimization of the complete Cd–Pr system based on relevant literature data on phase equilibria and thermodynamic properties, and supported by additional experimental values for the enthalpy of formation of the intermetallic compounds Cd_2_Pr, Cd_3_Pr, Cd_58_Pr_13_ and Cd_6_Pr.

## 2 Literature overview

The Cd–Pr phase diagram has been investigated in detail by Reichmann et al. [11] who applied conventional methods, i.e. powder X-ray diffraction (XRD), differential thermal analysis (DTA), and scanning electron microscopy (SEM), to clarify the phase relationships, including extent of solid solutions, homogeneity ranges and isothermal reaction temperatures in the whole composition range. In addition, a complete literature survey concerning the intermetallic compounds observed within this system was given there. All intermetallic compounds, i.e. CdPr, Cd_2_Pr, Cd_3_Pr, Cd_45_Pr_11_, Cd_58_Pr_13_, Cd_6_Pr and Cd_11_Pr, presented previously by Gschneidner and Calderwood [12], were confirmed. Additionally, an extended solid solubility of 22.1 at% Cd in the high-temperature allotropic modification of Pr was reported. Apparently, the addition of Cd stabilizes the high-temperature modification β-Pr down to 450 °C where it decomposes in a eutectoid reaction. All isothermal reactions as well as the corresponding reaction temperatures relevant for the present CALPHAD-type optimization are listed in Table 1. All intermetallic compounds except Cd_6_Pr and Cd_11_Pr show noticeable homogeneity ranges in the order of ~1 at% which were defined by Reichmann et al. in their recent phase diagram study [11] in agreement with the results from vapour pressure measurements [10].

**Table 1:** All invariant reactions and respective phase compositions determined experimentally by [11] together with the calculated reaction temperatures for comparison.

Reaction	*T* (°C)		Phase compositions (at% Cd)						Reaction type
	Calculated	Experimental	Calculated			Experimental			
aL+Cd_11_Pr⇄Cd	321	322	~100	~100	91.7	~100	91.7	~100	Degenerate peritectic
L+Cd_6_Pr⇄Cd_11_Pr	570	566	97.2	85.7	91.7	96.5	85.7	91.7	Peritectic
L+Cd_58_Pr_13_⇄Cd_6_Pr	734	740	92.4	81.7	85.7	90.5	81.8	85.7	Peritectic
L ⇄ Cd_58_Pr_13_	876	870		81.7			81.7		Congruent melting
Cd_58_Pr_13_+Cd_3_Pr⇄Cd_45_Pr_11_	800	795	81.7	75.0	80.4	80.4	76.3	79.8	Peritectoid
L⇄Cd_58_Pr_13_+Cd_3_Pr	867	856	78.8	81.7	75.0	78.9	81.3	76.3	Eutectic
L⇄Cd_2_Pr	984	991		66.7			66.7		Congruent melting
L+α-Cd_2_Pr⇄Cd_3_Pr	869	863	78.8	66.7	75.0	78.5	67.0	76.3	Peritectic
L⇄CdPr	999	1003		50.0			50.0		Congruent melting
L⇄β-Cd_2_Pr+CdPr	947	940	59.2	66.7	50.0	59.1	65.2	50.1	Eutectic
L⇄CdPr+β-Pr	712	709	23.0	47.1	20.3	25.0	47.0	22.1	Eutectic
β-Pr⇄CdPr+α-Pr	437	450	17.0	47.4	4.5	16.8	47.1	4.0	Eutectoid

a Reaction was modelled as degenerate eutectic L ⇄Cd+Cd_11_Pr (compare text).

Besides the work of Reichmann et al. [11] only limited information concerning phase diagram data was available from literature. Johnson et al. [13] reported liquidus data in the composition range up to 1.83 at% Pr, determined by chemical analysis of filtered samples of the corresponding equilibrium phases. These data were considered in Ref. [11]. In addition, Johnson et al. applied DTA and presented at least two invariant reactions. They argued for a degenerate eutectic reaction between Cd and Cd_11_Pr and a peritectic decomposition of Cd_11_Pr at 570 °C, at which temperature 3.5 at% Pr are soluble in liquid Cd. In their DTA measurements Reichmann et al. could show that the degenerate reaction between Cd and Cd_11_Pr must actually be a peritectic reaction. However, the peritectic formation temperature of Cd_11_Pr agrees quite well with the results of Ref. [11] where it was re-determined as 566 °C.

In a previous paper Veleckis and Van Deventer [14] determined experimentally an invariant reaction temperature of 435 °C for a eutectic reaction between Pr and CdPr, a value which corresponds obviously to the eutectoid decomposition reaction of β-Pr (see above). It must be assumed that Veleckis and Van Deventer did not consider the high-temperature allotropic modification of Pr.

As far as thermodynamic information is concerned, Reichmann and Ipser [10] determined Cd vapour pressures as a function of composition and temperature, using an isopiestic vapour pressure method. From these data the authors derived activity values of Cd at 823 K. By using an activity value for Pr in the two-phase field Cd_11_Pr+L, taken from Johnson and Yonco [15], as integration constant, a Gibbs–Duhem integration was performed to calculate Gibbs energies of formation at 823 K. In the study by Johnson and Yonco, the Gibbs energy of formation of the compound Cd_11_Pr had been determined by means of a Gibbs–Duhem integration based on thermodynamic activity values of Pr in Cd_11_Pr from own emf measurements. They showed that both, enthalpy and entropy of formation, of Cd_11_Pr were independent of temperature between 635 and 825 K.

In an early work by Castrillejo et al. [16], Gibbs energies of formation of the three intermetallic compounds Cd_11_Pr, Cd_6_Pr and Cd_58_Pr_13_ were measured by electrochemical techniques. The corresponding values at 823 K were determined to be −11.2±0.1, −18.7±0.1 and −22.9±0.1 kJ mol(at)^−1^. Comparing these values with Gibbs energies of formation given by Reichmann and Ipser [10] a quite good agreement can be observed. In addition, Castrillejo et al. listed partial Gibbs energies of Pr in the two-phase fields Cd_58_Pr_13_+Cd_6_Pr, Cd_6_Pr+Cd_11_Pr, and Cd_11_Pr+L, given as −107.6±0.6, −127.4±0.9 and −133.8±1.2 kJ mol(at)^−1^ for 823 K.

Based on the results of Johnson and co-workers [13], [15], Kurata and Sakamura [17] made a CALPHAD-type optimization of the Cd–Pr system up to 25 at% Pr. They considered Cd_11_Pr, Cd_6_Pr and Cd_58_Pr_13_ as line-compounds in their calculations and defined temperature dependent Gibbs energies for two of these compounds. Moreover, Kurata and Sakamura presented activity values of Pr in liquid Cd derived from their calculations.

Recently, experimental heat capacities became available for the compound Cd_11_Pr [24]; they had been obtained for the temperature interval 300–550 K by differential scanning calorimetry (DSC).

## 3 Experimental

To provide additional input data for the present CALPHAD-type optimization, calorimetric measurements were performed in two ways. Reaction calorimetric measurements were carried out in Genova to determine the enthalpy of formation of the intermetallic compound Cd_58_Pr_13_. In addition, solution calorimetry was used in Vienna to obtain enthalpies of formation of Cd_2_Pr, Cd_3_Pr, Cd_58_Pr_13_ and Cd_6_Pr. The experimental setups of both methods are discussed in detail in 3.1, 3.2. All calorimetric results are listed in Table 2.

**Table 2:** Standard enthalpies of formation measured by reaction and solution calorimetry; estimated error ±2 kJ/mol(at); reference state: Cd(s), α-Pr(s).

Phase	Solution calorimetry Δ_*f*_*H*/kJ mol(at)^−1^	Reaction calorimetry Δ_*f*_*H*/kJ mol(at)^−1^
Cd_6_Pr	−24.7	–
Cd_58_Pr_13_	−34.3^a^	−36.0^a^
Cd_3_Pr	−38.8	–
Cd_2_Pr	−46.0	–

a Average of two measured values, see Section 3.

### 3.1 Reaction calorimetry

High purity Cd rods (5N, Koch-Light Laboratories LTD., Colnbrook, England) and Pr pieces (99.9%, Smart Elements, Vienna, Austria) were used for sample preparation. To lower diffusion paths and increase the reaction rate during the calorimetric synthesis, samples were prepared with Cd and Pr powders. The metals were filed inside a glove box under Ar atmosphere (oxygen level: <1 ppm, water level: <1 ppm) to prevent oxidation. The metal powders were then weighed according to calculated amounts, mixed homogeneously and pressed into compact pellets. For the calorimetric measurements, the pellets were enclosed in tight-sealed Ta crucibles to prevent oxidation and to avoid possible weigh-losses due to the rather high vapour pressure of Cd.

All calorimetric experiments were performed using a high temperature drop calorimeter described repeatedly in literature [18], [19]. Heat effects were evaluated following a series of calibration runs by dropping specimens of known heat content, typically pure Ag, into the calorimeter. Each measurement involved two separate runs: a reaction run and a reference run; a detailed description is given by Ghosh et al. [20]. All runs were performed at a drop temperature of 298 K and a calorimeter temperature of 1048 K. In the first run, the so-called reaction run, the sample is dropped into the calorimeter, and the observed heat effect Δ*H*_1_ is due to

(1) $$\text{Cd}(s,\;298\;K)+\mathrm{Pr}(s,\;298\;K)\to {\mathrm{Cd}}_{58}{\mathrm{Pr}}_{13}(s,\;1048\;K)$$

After retrieving the reacted sample from the calorimeter, it is dropped once more into the calorimeter where the following heat effect Δ*H*_2_ is observed:

(2) $${\mathrm{Cd}}_{58}{\mathrm{Pr}}_{13}(s,\;298\;K)\to {\mathrm{Cd}}_{58}{\mathrm{Pr}}_{13}(s,\;1048\;K)$$

By taking the difference Δ*H*_1_−Δ*H*_2_, crucible effects are cancelled out and the enthalpy of formation of Cd_58_Pr_13_ is observed at 298 K

(3) $$\Delta H_{1}-\Delta H_{2}=\Delta_{f}H_{{\mathrm{Cd}}_{58}{\mathrm{Pr}}_{13}}^{0}(s,\;298\;K)$$

The equilibrium state of every sample after the reaction run was checked by standard phase analysis methods (LOM, SEM, X-ray powder diffraction, EPMA). The respective uncertainty of the enthalpy of mixing values is estimated to be around ±2 kJ/mol(at). The accuracy of the measurement and the evaluation of the error are discussed in detail in Refs. [18], [19], [20].

### 3.2 Solution calorimetry

Solution calorimetry in liquid Sn was performed using a Calvet-type twin calorimeter with two thermopiles with more than 200 thermocouples each. Enthalpies of formation were measured indirectly by dropping pure Cd (99.9999% Alfa AESAR, Karlsruhe, Germany), Pr (99.9%, Smart Elements, Vienna, Austria) and corresponding pieces of the intermetallic compounds into molten Sn at 823 °C. The intermetallic compounds were synthesized in their stoichiometric ratios using an isothermal isopiestic method according to Ref. [10]. All samples were determined to be pure single-phase by powder-XRD. An automated drop device was used and drops were performed under an Ar atmosphere. Ten sample pieces (between 30 and 50 mg each) and additional five pieces of NIST standard sapphire, for the calibration of the heat signal, were dropped at each calorimetric run. Graphite was used as crucible material. No reaction between the metals and the crucible material was observed in any measurement. Limiting heats of solution $\Delta_{\mathrm{sol}}{\overset{¯}{H}}_{i}^{0}$ were derived by extrapolation for Cd, Pr and the respective compounds and enthalpies of formation were evaluated according to

(4) $$\Delta_{f}H_{{\mathrm{Cd}}_{x}{\mathrm{Pr}}_{y}}^{T_{d}}=x\Delta_{\mathrm{sol}}{\overset{¯}{H}}_{\mathrm{Cd}}^{0}+y\Delta_{\mathrm{sol}}{\overset{¯}{H}}_{\mathrm{Pr}}^{0}-\Delta_{\mathrm{sol}}{\overset{¯}{H}}_{{\mathrm{Cd}}_{x}{\mathrm{Pr}}_{y}}^{0}$$

where *T*_d_ is the drop temperature (298 K). The furnace temperature and the drop temperature were recorded for each drop; the calculations were made using mean values over all drops. The scattering of the temperature signals were low and did not influence the measurements significantly.

Besides all instrumental errors, systematic errors were estimated to be mainly due to incomplete mixing of the samples with the solvent as well as due to evaporation of Cd. In general it was observed that some Cd condensed at the colder part of the silica glass tubes during the calorimetric runs. Therefore, the intervals between individual drops were reduced to 40 min. Although the effect was still present it was clearly less significant. According to the evaluation of the results of these measurements (compare chapter 4), the overall error for the enthalpies of formation is estimated to be ±2 kJ/mol(at).

## 4 Thermodynamic modelling

The aim of the present work was to derive a set of thermodynamic model parameters, describing Gibbs energies of all phases in the system, which can be used as input data for a CALPHAD-type calculation. The optimization, described in detail by Lukas et al. [21], is based on obtaining the minimum total Gibbs energy of the system at constant temperature and pressure, yielding the composition and the amount of phases in equilibrium. For the calculations themselves the ThermoCalc® Classic software package (version S) [25] was used.

### 4.1 Pure elements

The Gibbs energy function Giθ0=Giθ−HiSER for element *i* (*i*=Cd, Pr) in the phase *θ* (*θ*=α-Pr, β-Pr, Cd, or liquid) is described by a power series as defined by Dinsdale [26]. The corresponding parameters were taken from the SGTE 5.1 database included in the software package.

### 4.2 Modelling of liquid and solid solution phases

The solid solutions based on α-Pr and β-Pr as well as the liquid phase were modelled in terms of a standard substitutional model with one sublattice. The molar Gibbs energy of a solution phase *θ* is described as follows:

(5) $$G_{m}^{\theta }=G_{\mathrm{ref}}^{\theta }+G_{\mathrm{id}}^{\theta }+G_{E}^{\theta }+G_{\mathrm{other}}^{\theta }+...$$

where $G_{\mathrm{ref}}^{\theta }$ is the molar Gibbs energy of the weighted sum of the system constituents *i* in the crystallographic structure corresponding to the phase *θ* relative to the chosen reference state (typically the stable element reference state, SER)

(6) Grefθ=∑i=1nxiGiθ0

and its temperature dependence is given by

(7) $$G(T)=a+bT+cT\;\ln(T)+\sum_{n}d_{n}T^{n}$$

where *a*−*d*_i_ are adjustable coefficients.

The contribution to the Gibbs energy from ideal random mixing of the constituents in the crystal lattice or in the liquid, denoted $G_{\mathrm{id}}^{\theta }$, is defined as ideal mixing

(8) $$G_{\mathrm{id}}^{\theta }=RT\sum_{i=1}^{n}x_{i}\;\ln(x_{i})$$

for a system consisting of *n* components.

The Gibbs energy which describes the effect of non-ideal mixing behaviour on the thermodynamic properties of a solution phase is given by the usual Redlich–Kister formalism [22]

(9) GEθ=xixj(Lij0+Lij1(xi−xj)+Lij2(xi−xj)2+...)

where the temperature-dependent interaction parameters, describing the mutual interaction between constituents *i* and *j*, are denoted ^*ν*^*L* (*ν*=0, 1, 2, …). The temperature dependence of the interaction parameters is usually defined as

(10) Lijν=aν+bνT

### 4.3 Modelling of intermetallic phases

The intermetallic phase CdPr with CsCl-(B2-) structure was modelled with a two-sublattice model (Pr)_0.5_(Cd,Pr)_0.5_; since the experimental results indicate a homogeneity range to the Pr-rich side only, it was assumed that the Pr-sublattice remains fully occupied whereas on the Cd-sublattice a small amount of Cd-atoms can be substituted by Pr-atoms. This was discussed in detail in Ref. [11]. The $G_{\mathrm{ref}}^{\theta }$ for such a model is given by

(11) Grefθ=∑yi1yj2G(i:j)0i,j=Cd,Pr

where the *y* terms are the site fractions of each constituent in the relevant sublattices, 1 and 2. The term G(i:j)0 is the Gibbs energy of formation of the “virtual compound” (or “end member”) *ij*.

All other phases were treated as stoichiometric compounds, i.e. no variability concerning the composition was considered. The Gibbs energy is modelled relative to the surface of reference

(12) Gmθsrf=∑i=1nbiGiSER

with $G_{i}^{\mathrm{SER}}$ as the Gibbs energy of component *i* in the stable element form and *b*_*i*_ as the stoichiometric coefficient for *i* in the phase *θ*.

## 5 Results and discussion

### 5.1 Calorimetric measurements

The enthalpies of formation at the stoichiometric compositions of Cd_2_Pr, Cd_3_Pr, Cd_58_Pr_13_ and Cd_6_Pr, derived from the present calorimetric measurements, are listed in Table 2. All values are referred to the standard reference states of Cd and Pr and are given at 298 K.

Reaction calorimetric measurements were done by dropping samples with the stoichimetric composition Cd_58_Pr_13_ into the calorimeter. Additionally, samples with compositions close to Cd_58_Pr_13_ were dropped to obtain the trend of Δ_f_*H*^0^ vs. composition, and to evaluate the most reliable value. From these experiments, two values were derived at the stoichiometric composition of Cd_58_Pr_13_, namely −35.0 kJ/mol(at) and −37.0 kJ/mol(at), and the corresponding accuracy was calculated to be within ±1.4 kJ/mol(at). Thus, an average value of −36.0 kJ/mol(at) is given in Table 2. Considering all possible statistical and systematic errors, as outlined in detail by Delsante and Borzone [23], the resulting error should not exceed ±2 kJ/mol(at).

From solution calorimetry, enthalpies of formation could be derived for Cd_2_Pr, Cd_3_Pr, Cd_58_Pr_13_ and Cd_6_Pr. The rather Cd-rich compound Cd_58_Pr_13_ was measured twice which allowed the estimation of an internal error for the present measurements. The two values, i.e. −35.1 kJ/mol(at) and −33.4 kJ/mol(at) with an error of ±2 kJ/mol(at), were found to be in good agreement with each other. Again, an average value of 34.4 kJ/mol(at) is listed in Table 2. Comparing the results of the two different calorimetric methods, a value of −35±2 kJ/mol(at) is suggested for the standard enthalpy of formation of Cd_58_Pr_13_.

**Fig. 1:**
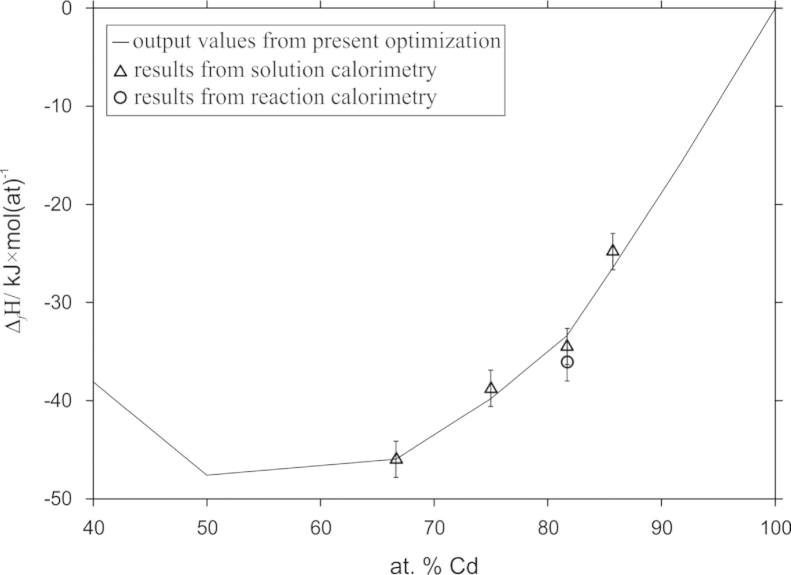
Comparison of enthalpies of formation from calorimetric measurements with output values from the present calculation; reference state: Cd(s) and α-Pr(s); error bars are given according to chapter 3.

The accuracy of the enthalpy of formation for Cd_2_Pr, Cd_3_Pr and Cd_6_Pr was assumed to be similar and is given likewise with ±2 kJ/mol(at). The corresponding enthalpy values, together with the results of the CALPHAD optimization, are presented in Fig. 1. As it was indicated already earlier by Reichmann and Ipser [10], an exothermic behaviour is observed within the composition range 40–100 at% Cd.

### 5.2 Thermodynamic optimization

The present optimization is based on thermodynamic data and phase diagram information collected for this system. Gibbs energies of formation and activity values of Cd at 823 K were taken from Reichmann and Ipser [10]. Additionally, enthalpies of formation from the present study were used as input values. The phase diagram was optimized according to the experimentally determined version published by Reichmann et al. [11] and liquidus values from Johnson et al. [13]. All additional data were taken for comparison.

**Fig. 2:**
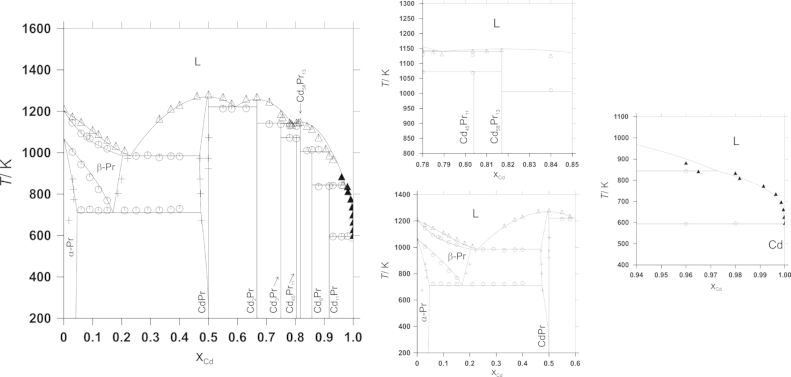
Comparison of the calculated Cd–Pr phase diagram with data available from literature; open triangles and circles: DTA values, plus signs: SEM data, both from Reichman et al. [11]; filled triangles: Johnson et al. [13].

The calculated phase diagram is shown in Fig. 2 together with experimental results. A comparison between calculated and experimentally determined invariant reaction temperatures is listed in Table 1. The entire composition range could be well described with the present model. Considering the rather small homogeneity ranges of the compounds (compare Refs. [10], [11]) only CdPr was introduced with some solubility into the model while all other compounds were treated as line compounds. According to the results by Reichmann et al. [11], CdPr dissolves apparently around 3 at% Pr by substituting Cd sites. The corresponding model parameters are given in Table 3.

**Table 3:** All parameters of the thermodynamic assessment of the Cd–Pr phase diagram are given for the temperature interval 298–1600 K in Joules.

DHCP (α-Pr):
^0^*G*_Cd_=*GHSERCD*+5000
^0^*L*_Cd,Pr_=−108290+60*T*
BCC (β-Pr):
^0^*L*_Cd,Pr:Va_=−152571+68*T*
^1^*L*_Cd,Pr:Va_=−54630+23*T*
LIQ:
^0^*L*_Cd,Pr_=−132020+52*T*
^1^*L*_Cd,Pr_=−27000+1*T*
^2^*L*_Cd,Pr_=−2000+1*T*
Cd_11_Pr
^0^*G*_Cd:Pr_=0.917·*GHSERCD*+0.083·*GHSERPR*
−15900+3*T*
Cd_6_Pr
^0^*G*_Cd:Pr_=0.857·*GHSERCD*+0.143·*GHSERPR*
−26502.7+6.6*T*
Cd_58_Pr_13_
^0^*G*_Cd:Pr_=0.817·*GHSERCD*+0.183·*GHSERPR*
−33366.4+9.5*T*
Cd_45_Pr_11_
^0^*G*_Cd:Pr_=0.804·*GHSERCD*+0.196·*GHSERPR*
−34708.7+10.126*T*
Cd_3_Pr
^0^*G*_Cd:Pr_=0.75·*GHSERCD*+0.25·*GHSERPR*
−39827.2+12.3*T*
Cd_2_Pr
^0^*G*_Cd:Pr_=0.667·*GHSERCD*+0.333·*GHSERPR*
−45989.7+14.2*T*
CdPr
^0^*G*_Cd:Pr_=0.5·*GHSERCD*+0.5·*GHSERPR*
−47613.2+15.7*T*
^0^*G*_Pr:Pr_=*GHSERPR*+6180.5+3*T*

A temperature dependent phase transformation of Cd_2_Pr, suggested in Ref. [11], was not considered in the calculations but the two modifications of Cd_2_Pr were treated as one single phase. In Ref. [11] there were strong experimental indications for a degenerate peritectic formation reaction of (Cd) at 322 °C. To simplify the calculations this was considered as a degenerate eutectic reaction in the present study. Treating the reaction as a peritectic would be only possible when assuming some solubility of Pr in Cd. Since no experimental information on solubility is available, this was avoided here.

**Fig. 3:**
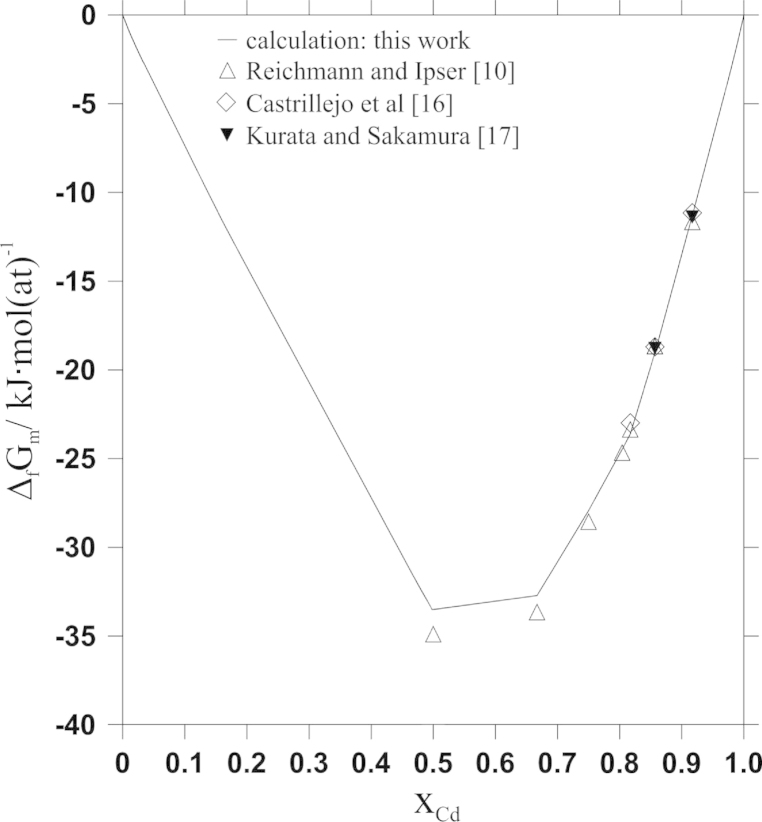
Comparison of calculated Gibbs energies of formation at 823 K with values from literature; reference states: Cd(l) and α-Pr(s).

**Fig. 4:**
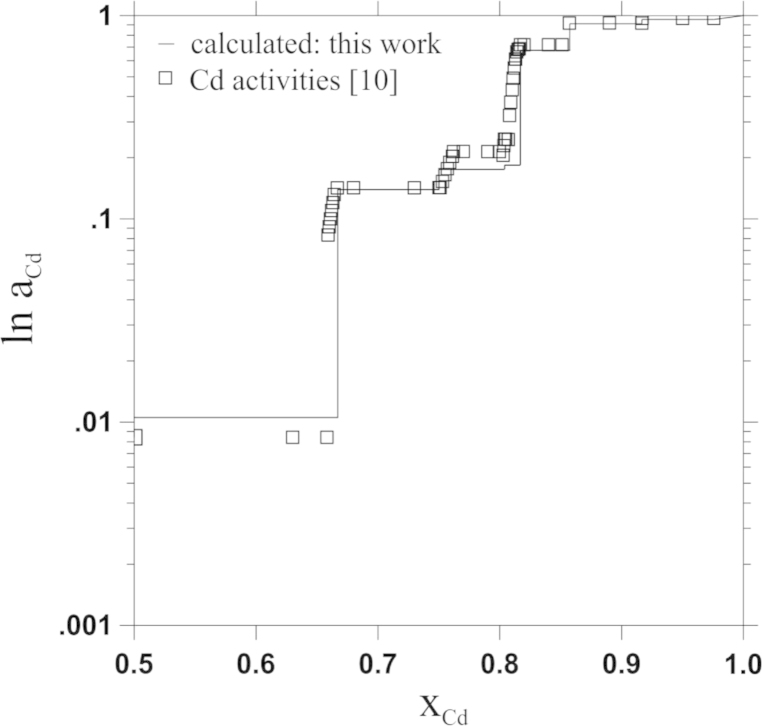
Comparison of calculated Cd activities with values from literature; 823 K, and reference state: Cd(l).

All line-compounds were modelled using temperature dependent contributions to the total Gibbs energies. The respective thermodynamic parameters are listed in Table 3. Experimental Gibbs energies of formation at 823 K served as starting points for the modelling and the parameters *a* and *b* for all stoichiometric phases were subsequently optimized according to experimental thermodynamic data. Since no thermodynamic information was available for the liquid, the parameters for the liquid were subsequently adapted to reproduce the experimental phase diagram data. Calculated Gibbs energies of formation at 823 K are shown together with values from literature in Fig. 3; it can be seen that the overall agreement is quite good. Similar as for the Gibbs energies of formation, it was possible to adjust Cd activities to the values determined experimentally by Reichmann and Ipser [10]. Again, the comparison presented in Fig. 4 shows very good agreement. Nevertheless, some deviations between calculated and experimentally determined Cd activities occurred in the two-phase fields adjacent to Cd_45_Pr_11_. Considering that the stoichiometric compositions of Cd_45_Pr_11_ and Cd_58_Pr_13_ are very close to each other, it was reasonable to model them with a similar temperature dependence of the energy contribution. Only in this way it was possible to model both compounds stable in the whole temperature range. The values shown in Fig. 4 lead to the best consistency of all input data.

As described above, the modelled Gibbs energies of formation for Cd_6_Pr, Cd_58_Pr_13_, Cd_3_Pr and Cd_2_Pr are based on the enthalpy values from the present calorimetric measurements, compare Table 2. The respective calculated enthalpies of formation were compared with those determined experimentally (Fig. 1). Considering the accuracy of ±2 kJ/mol(at) of the calorimetric measurements, the respective calculated enthalpies of formation are within the error limit.

**Fig. 5:**
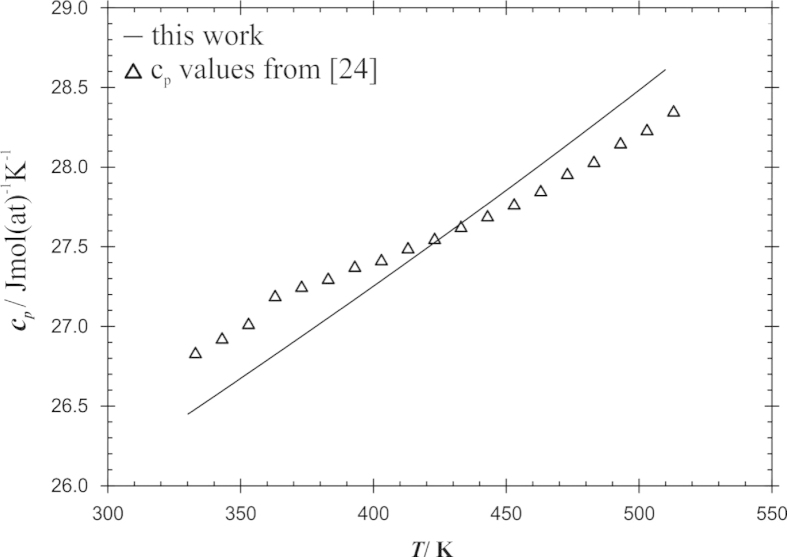
Heat capacity of Cd_11_Pr between 300 and 550 K; line: values according Neumann–Kopp from the present calculation, triangles: data determined with DSC [24].

As can be observed in Table 3, only the parameters *a* and *b* were modelled for the individual Gibbs energies of the intermetallic compounds, following the Neumann–Kopp rule. C_p_ data of Cd_11_Pr were measured by DSC by Kumar et al. [24] between 300 and 550 K. The comparison with the calculated values, shown in Fig. 5, indicates that the deviation of the heat capacity of Cd_11_Pr from Neumann–Kopp׳s rule is minimal (within the experimental error) and does not require the introduction of higher order terms to the Gibbs energy description.

The intermetallic compound CdPr was modelled with an ideal two-sublattice model. By modelling of the hypothetical end member GPr:Pr0, using a temperature dependent contribution, the homogeneity range of CdPr was optimized according to the experimental data.

The solid solutions of Cd in the low- (*DHCP*, α-Pr) and high-temperature (*BCC*, β-Pr) modifications of Pr were modelled according to the Redlich–Kister formalism using one (^0^*L*) and two (^0^*L*, ^1^*L*) interaction parameters, respectively. In order to describe the experimentally determined liquidus along the whole composition range with the present model, three interaction parameters ^0^*L*, ^1^*L* and ^2^*L* were required for the liquid phase.

It should be pointed out that the present thermodynamic optimization is exclusively valid for the temperature range between 298 and 3200 K and does not include temperatures above and below.

## 6 Summary

Standard enthalpies of formation of the four intermetallic compounds Cd_6_Pr, Cd_58_Pr_13_, Cd_3_Pr and Cd_2_Pr were measured by calorimetry. Solution calorimetry was employed for all four compounds and the enthalpy of formation of stoichiometric Cd_58_Pr_13_ was also measured by direct reaction calorimetry. The experimental values for Cd_58_Pr_13_ from the two different methods were in good agreement with each other, and an average value of −35±2 kJ/mol(at) is suggested. All present calorimetric data together with thermodynamic data from literature served as input data for a thermodynamic assessment of the Cd–Pr phase diagram. The complete composition range including all invariant reactions could be calculated, and the agreement with phase diagram data presented by Refs. [11], [13] is very good. A comparison of calculated and experimentally determined phase diagram is given in Fig. 2 and Table 1. A temperature dependent contribution to the individual Gibbs energy was introduced for all phases to guarantee a consistent description of all input data.
